# A Phenotypically Silent *vanB2* Operon Carried on a Tn*1549*-Like Element in *Clostridium difficile*

**DOI:** 10.1128/mSphere.00177-16

**Published:** 2016-08-10

**Authors:** Daniel R. Knight, Grace O. Androga, Susan A. Ballard, Benjamin P. Howden, Thomas V. Riley

**Affiliations:** a Microbiology and Immunology, School of Pathology and Laboratory Medicine, The University of Western Australia, Nedlands, Western Australia, Australia; b Microbiological Diagnostic Unit Public Health Laboratory and Doherty Centre for Applied Microbial Genomics, Department of Microbiology and Immunology, The Peter Doherty Institute for Infection and Immunity, The University of Melbourne, Parkville, Victoria, Australia; c Department of Microbiology, PathWest Laboratory Medicine, Queen Elizabeth II Medical Centre, Nedlands, Western Australia; d School of Medical and Health Sciences, Edith Cowan University, Joondalup, Western Australia, Australia; e School of Veterinary and Life Sciences, Murdoch University, Murdoch, Western Australia, Australia; Centers for Disease Control and Prevention

**Keywords:** antimicrobial resistance, *Clostridium difficile* infection, mobile genetic element, *vanB*

## Abstract

In the last decade, *Clostridium difficile* infection (CDI) has reached an epidemic state with increasing incidence and severity in both health care and community settings. Vancomycin is an important first-line therapy for CDI, and the emergence of resistance would have significant clinical consequences. In this study, we describe for the first time a *vanB2* vancomycin resistance operon in *C. difficile*, isolated from an Australian veal calf at slaughter. The operon was carried on an ~42-kb element showing significant homology and synteny to Tn*1549*, a conjugative transposon linked with the emergence and global dissemination of vancomycin-resistant enterococci (VRE). Notably, the *C. difficile* strain did not show any reduced susceptibility to vancomycin *in vitro* (MIC, 1 mg/liter), possibly as a result of an aberrant *vanRB* gene. As observed for other anaerobic species of the animal gut microbiota, *C. difficile* may be a reservoir of clinically important vancomycin resistance genes.

**IMPORTANCE** In an era when the development of new antimicrobial drugs is slow, vancomycin remains the preferred antimicrobial therapy for *Clostridium difficile* infection (CDI), the most important health care-related infection in the world today. The emergence of resistance to vancomycin would have significant consequences in relation to treating patients with CDI. In this paper, we describe for the first time a complete set of vancomycin resistance genes in *C. difficile*. The genes were very similar to genes found in vancomycin-resistant enterococci (VRE) that were associated with the emergence and global dissemination of this organism. Fortunately, the *C. difficile* strain did not show any reduced susceptibility to vancomycin *in vitro* (MIC, 1 mg/liter), possibly because of a small difference in one gene. However, this observation signals that we may be very close to seeing a fully vancomycin-resistant strain of *C. difficile*.

## Abstract (precis)

In an era when the development of new antimicrobial drugs is slow, vancomycin remains the preferred antimicrobial therapy for *Clostridium difficile* infection (CDI), the most important health care-related infection in the world today. The emergence of resistance to vancomycin would have significant consequences in relation to treating patients with CDI. In this paper, we describe for the first time a complete set of vancomycin resistance genes in *C. difficile*. The genes were very similar to genes found in vancomycin-resistant enterococci (VRE) that were associated with the emergence and global dissemination of this organism. Fortunately, the *C. difficile* strain did not show any reduced susceptibility to vancomycin *in vitro* (MIC, 1 mg/liter), possibly because of a small difference in one gene. However, this observation signals that we may be very close to seeing a fully vancomycin-resistant strain of *C. difficile*.

## Observation

Since its first description as the causative agent of pseudomembranous colitis in 1978, *Clostridium difficile* has emerged as a major enteropathogen of humans and a significant burden to global health care systems (1). Vancomycin has been a first-line therapy for *C. difficile* infection (CDI) for almost 30 years, retaining good activity against *C. difficile*, including strains belonging to epidemic lineages and those with increased resistance to metronidazole (2). Despite sporadic reports of reduced susceptibility to vancomycin (MIC, ≥4 mg/liter; CLSI susceptibility breakpoint, ≤2 mg/liter), to date no underlying mechanisms have been identified (2). Sequencing of *C. difficile* genomes revealed the widespread presence of a vancomycin resistance operon (*vanG_Cd_*) (3). Although it is often referred to as cryptic (phenotypically silent), transcriptional and biochemical studies showed that *vanG_Cd_* was functional, conferring a modest increase in MIC in *C. difficile* (from 1 mg/liter to 2 mg/liter) (4). Here, we present the first description of a cryptic *vanB2* operon in *C. difficile*, carried on an ~42-kb element showing significant homology and synteny to Tn*1549*, a conjugative transposon (CTn) linked with the emergence and global dissemination of vancomycin-resistant enterococci.

*C. difficile* strain AI0499 was recovered from the carcass of a calf (aged <7 days) in Victoria, Australia, in April 2013, identified as *C. difficile* by morphological and phenotypic traits as previously described (5), and confirmed by matrix-assisted laser desorption ionization–time of flight (MALDI-TOF) mass spectrometry. By PCR ribotyping, strain AI0499 was identified as ribotype (RT) 033 and thus negative for genes encoding large clostridial toxins A and B (*tcdA*^−^ *tcdB*^−^) but positive for genes encoding binary toxin (*cdtA^+^ cdtB^+^*) (5).

Whole-genome sequencing (WGS) of AI0499 was performed in duplicate at two independent institutions. Genomic DNA (gDNA) was extracted using a Gentra Puregene kit (Qiagen, Hilden, Germany), and libraries were created using Nextera XT protocols (Illumina, Inc., San Diego, CA). The first sequence run was performed on an Illumina MiSeq sequencer with 250-bp paired-end (PE) chemistry, generating 406,204 reads and 36× coverage. The second was performed on an Illumina HiSeq sequencer with 100-bp PE chemistry, generating 3,684,407 reads and 131× coverage. Multilocus sequence typing (MLST) and antimicrobial gene profiling were performed using SRST2 (6). Genomes were assembled, annotated, and curated using a pipeline comprising SPAdes, Prokka, Artemis, and Easyfig (7–10). *In vitro* susceptibility to vancomycin was investigated in triplicate using the CLSI agar dilution methodology as previously described (11) and in triplicate using Etest methodology.

WGS and *de novo* assembly of the AI0499 genome revealed a single chromosome of 4,095,918 bp and 28.75% GC with 3,960 coding sequences (CDS) and an overall coverage of ~130×. Strain AI0499 was characterized as sequence type (ST) 11 (MLST clade 5) and harbored a complete binary toxin locus comprising *cdtR*, *cdtA*, and *cdtB* genes. Strain AI0499 possessed an uncommon pathogenicity locus identified as toxinotype XI and defined by the complete absence of *tcdB*, a fragmented and truncated *tcdA* gene (A2 fragment, 3,231 bp; A3 fragment, 915 bp), and a variant *tcdC* gene (allele *tcdC*-A1 as described by Curry et al.) (12, 13). SRST2 identified seven vancomycin resistance genes with >99% sequence identity to *vanXB*, *vanB*, *vanHB*, *vanW*, *vanYB*, *vanSB*, and *vanRB*. AI0499 was negative for *vanG_Cd_*.

In Gram-positive bacteria, vancomycin resistance is mediated by several *van* operons and arises as a result of both (i) biosynthesis of modified peptidoglycan precursors ending in d-Ala-d-Lac or d-Ala-d-Ser to which vancomycin shows reduced binding and (ii) the elimination of high-affinity natural d-Ala-d-Ala precursors (13). Tn*1549* (GenBank accession no. AF192329) is a member of the Tn*916* family of conjugative transposons and harbors a *vanB* subtype 2 operon (*vanB2*) comprising genes encoding a dipeptidase (*vanXB*, 609 bp), a ligase (*vanB*, 1,029 bp), a dehydrogenase (*vanHB*, 972 bp), a putative hydrolase (*vanW*, 828 bp), and a carboxypeptidase (*vanYB*, 807 bp). Two further genes, *vanSB* (1,344 bp) and *vanRB* (663 bp), also colocated within the *vanB2* operon, play a crucial role in the phenotypic expression of vancomycin resistance (14).

**FIG 1:**
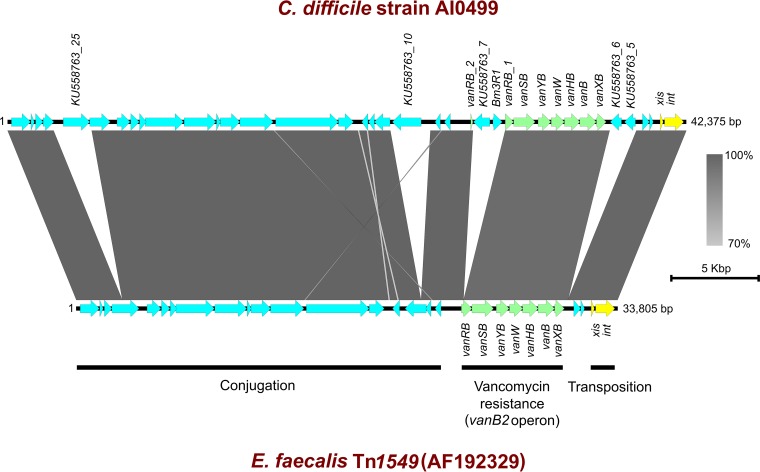
Comparative genomic analysis of Tn*1549*-like element in *C. difficile* strain AI0499 and prototypical Tn*1549* of *Enterococcus faecalis* (GenBank accession no. AF192329). Arrows indicate open reading frames (ORFs) and direction of transcription. Excisionase (*xis*) and integrase (*int*) genes are shown in yellow, and genes comprising the *vanB2* operon (*vanXB*, *vanB*, *vanHB*, *vanW*, *vanYB*, *vanSB*, and *vanRB*) are shown in green, with the remaining ORFs shown in blue. The figure was prepared using Easyfig (minimum blast hit length of 100 bp and a maximum E value of 0.001) (10). Vertical blocks between sequences indicate regions of homology with Blast nucleotide identity shown on a colored scale ranging from 70% (light gray) to 100% (dark gray). *Bm3R1* and ORFs KU558763_5, KU558763_6, KU558763_7, KU558763_10, and KU558763_25 are shown to be present in strain AI0499 but absent from the sequence with accession no. AF192329 with *Bm3R1* and KU558763_7 interrupting *vanRB* (*vanRB_1* and *vanRB_2* fragments shown). Overall sizes of elements in strain AI0499 and the sequence with accession no. AF192329 are 42,375 bp and 33,805 bp, respectively.

Sequence analysis of regions flanking the *vanB2* gene cluster in AI0499 revealed an element of 42,375 bp showing significant sequence identity and synteny with the prototypical Tn*1549* (GenBank sequence accession no. AF192329) (Fig. 1). Notably, this element differed markedly, particularly in its accessory region, from other putative Tn*1549*-like CTns, CTn*2*, CTn*4*, and CTn*5*, previously described in *C. difficile* (data not shown) (15, 16).

The element designated Tn*1549*-like contained 38 open reading frames (ORFs) and, like Tn*1549*, was organized into transposition, accessory (antimicrobial resistance), and conjugation regions (Fig. 1). Defining the left and right terminal ends of the element were 11-bp inverted repeats matching those found in Tn*1549* and likely representing excision/integration sites (14). Comparing the *vanB2* operon in AI0499 to that of Tn*1549* revealed significant homology in *vanXB*, *vanB*, *vanHB*, *vanW*, *vanYB*, and *vanSB* (Fig. 1)*.* However, in AI0499 *vanRB* was fragmented into a 525-bp fragment located adjacent to *vanSB* and a 134-bp fragment some 2.1 kb away (Fig. 1). Notably, two CDS present in strain AI0499 but absent in Tn*1549* were found interrupting the *vanRB* gene. *Bm3R1* (582 bp) and KU558763_7 (1,032 bp) encode a transcriptional repressor and decarboxylase originating from *Bacillus megaterium* and *Bacillus cereus*, respectively. The Tn*1549*-like element contained four additional CDS completely absent from Tn*1549* (Fig. 1). KU558763_5 (684 bp) and KU558763_6 (702 bp), colocated between the transposition and vancomycin resistance regions, encode hypothetical proteins originating from *Clostridium clostridioforme*. KU558763_10 (1,803 bp), located ~3 kb into the conjugation region, and KU558763_25 (1,665 bp), located near the far left extremity, both encode group II introns originating from an unidentified *Clostridiales* member and *C. clostridioforme*, respectively.

Several clostridial species, including *C. bolteae*, *C. hathewayi*, *C. innocuum*, *C. clostridioforme*, and *C. symbiosum*, harbor *vanB*-like elements and demonstrate vancomycin resistance *in vitro* (17–19). Notably, strain AI0499 did not show any reduced susceptibility to vancomycin *in vitro* (MIC, 1 mg/liter), most likely due to the fragmentation of *vanRB*; however, this first description of a phenotypically silent *vanB2* operon in *C. difficile* further confirms that anaerobes of the animal gut microbiota are a reservoir of clinically important *vanB-*like resistance operons.

### Accession number(s).

The nucleotide sequence of the Tn*1549*-like element from strain AI0499 has been submitted to GenBank (accession no. KU558763). The HiSeq PE sequence reads have been deposited in the NCBI Short Read Archive under accession no. SRP067713.
